# Impact of infectious disease outbreaks and regulatory policies on cord blood banking: a two-decade single-center analysis in Korea

**DOI:** 10.1093/stcltm/szag059

**Published:** 2026-08-05

**Authors:** Sue Shin, Nam-Hee Kim, Hye Ryun Lee, Jong Hyun Yoon, Byoung Jae Kim, Hyun-Woong Park, Eun Youn Roh

**Affiliations:** Department of Laboratory Medicine, Seoul Metropolitan Government–Seoul National University Boramae Medical Center, Seoul 07061, Republic of Korea; Seoul Metropolitan Government Public Cord Blood Bank (ALLCORD), Seoul 07061, Republic of Korea; Department of Laboratory Medicine, Seoul National University College of Medicine, Seoul 03080, Republic of Korea; Department of Laboratory Medicine, Seoul Metropolitan Government–Seoul National University Boramae Medical Center, Seoul 07061, Republic of Korea; Department of Laboratory Medicine, Seoul National University College of Medicine, Seoul 03080, Republic of Korea; Department of Laboratory Medicine, National Medical Center, Seoul 04564, Republic of Korea; Department of Laboratory Medicine, Seoul Metropolitan Government–Seoul National University Boramae Medical Center, Seoul 07061, Republic of Korea; Seoul Metropolitan Government Public Cord Blood Bank (ALLCORD), Seoul 07061, Republic of Korea; Department of Laboratory Medicine, Seoul National University College of Medicine, Seoul 03080, Republic of Korea; Seoul Metropolitan Government Public Cord Blood Bank (ALLCORD), Seoul 07061, Republic of Korea; Department of Obstetrics and Gynecology, Seoul Metropolitan Government–Seoul National University Boramae Medical Center, Seoul 07061, Republic of Korea; Department of Laboratory Medicine, Seoul Metropolitan Government–Seoul National University Boramae Medical Center, Seoul 07061, Republic of Korea; Department of Laboratory Medicine, Seoul National University College of Medicine, Seoul 03080, Republic of Korea; Department of Laboratory Medicine, Seoul Metropolitan Government–Seoul National University Boramae Medical Center, Seoul 07061, Republic of Korea; Seoul Metropolitan Government Public Cord Blood Bank (ALLCORD), Seoul 07061, Republic of Korea; Department of Laboratory Medicine, Seoul National University College of Medicine, Seoul 03080, Republic of Korea

**Keywords:** cord blood banking, public cord blood bank, total nucleated cell threshold, COVID-19 pandemic, regulatory policy, Korea, hematopoietic stem cell transplantation

## Abstract

Public cord blood (CB) banks face declining inventories worldwide, but the relative contributions of regulatory changes, declining birth rates, and infectious disease outbreaks remain unclear. We performed a retrospective analysis of 73 925 CB units submitted to a Korean public cord blood bank from 2006 to 2025, encompassing two regulatory transitions in total nucleated cell (TNC) criteria and three major outbreaks (H1N1, MERS, and COVID-19), using interrupted time series, correlation, and multivariable analyses. Of 73 925 submissions, 27 684 (37.4%) were banked. The 2011 TNC ≥ 8 × 10^8^ criterion produced a moderate reduction in banking rate from 37.7% to 33.6%, while the 2021 TNC ≥ 11 × 10^8^ revision caused an abrupt decline from 52.6% to 24.4%. H1N1 and MERS had minimal effects, whereas the COVID-19 period showed a sustained decline coinciding with the 2021 TNC revision. Birth decline correlated strongly with submissions (*R*^2^ = 0.94). Regulatory criteria changes were the primary driver of banking rate fluctuations, while declining births affected submission volume. Among three outbreaks, only the COVID-19 period overlapped with a sustained decline, likely reflecting concurrent regulatory change rather than the pandemic itself. CB quality and safety were maintained throughout all disruptions. These findings have direct translational implications for sustaining the global CB inventory as a critical source of hematopoietic stem cells for transplantation.

## Graphical Abstract

**Graphical Abstract:**
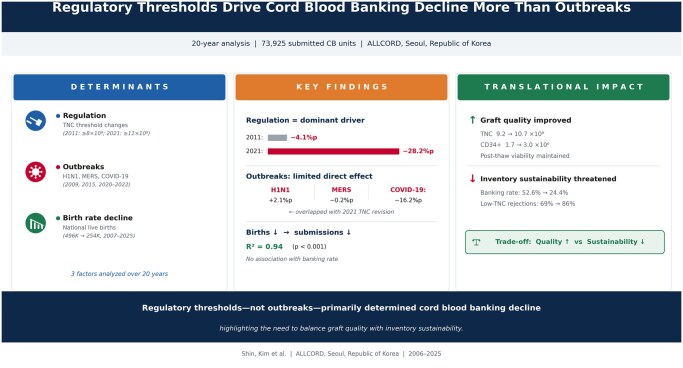

Significance statement Public cord blood banks provide a critical source of stem cells for transplantation, yet their inventories are declining worldwide. This study shows that regulatory quality thresholds, rather than infectious disease outbreaks, are the primary driver of this reduction. Although stricter criteria improve graft quality, they substantially reduce the proportion of bankable units. These findings highlight the need for policies that balance graft quality with the long-term sustainability of public CB inventories, ensuring continued access to this essential transplant resource.

## Clinical application/translation

This study demonstrates that regulatory thresholds, rather than infectious disease outbreaks, are the dominant determinant of cord blood banking dynamics. While stricter total nucleated cell (TNC) criteria improve graft quality, they substantially reduce banking rates and inventory size. These findings have direct clinical implications, as reduced inventory diversity may limit donor availability, particularly for patients lacking matched donors.

From a translational perspective, our results support the need for adaptive, data-driven regulatory strategies that balance graft quality with sustainable inventory levels. Such approaches may help optimize cord blood availability and ensure continued access to hematopoietic stem cell transplantation, especially in low-fertility settings.

## Introduction

Public cord blood (CB) banking provides a vital source of hematopoietic stem cells (HSCs) for allogeneic transplantation.^1^^,^^2^ Since the first successful CB transplantation, performed in 1988 and reported in 1989,^3^ more than 800 000 units have been cryopreserved in public CBBs worldwide.^4^ However, many CB banks now report declining inventories, threatening the sustainability of this transplant resource.^5^^,^^6^

Multiple factors contribute to this decline. Increasingly stringent regulatory criteria, particularly minimum total nucleated cell (TNC) count thresholds, reduce the proportion of bankable units.^7^^,^^8^ Declining birth rates in developed nations shrink the donor pool,^9^ while the emergence of haploidentical transplantation has provided alternative donor options.^10^^,^^11^ Additionally, infectious disease outbreaks may disrupt the CB supply chain—from donor recruitment to collection, processing, and distribution.^12^^,^^13^

The Republic of Korea offers a compelling model for studying these dynamics. Korea enacted its Cord Blood Management and Research Act in 2010 (enforced July 2011),^14^ set TNC criteria at ≥8 × 10^8^ (2011) and raised them to ≥11 × 10^8^ (2021), while experiencing an approximately 49% decline in live births (from 496 000 in 2007 to 254 500 in 2025).^9^ Korea also experienced three major outbreaks: the 2009 H1N1 pandemic, the 2015 MERS outbreak (186 cases, 38 deaths),^15^ and the 2020-2022 COVID-19 pandemic.^16^ Throughout this period, the bank actively supported clinical use, with a total of 781 units provided for transplantation.

Public cord blood banking in Korea is governed by the Act on Cord Blood Management and Research under the primary authority of the Ministry of Health and Welfare (MOHW). Review and evaluation of cord blood banks are delegated under the Enforcement Decree to the National Institute of Organ, Tissue and Blood Management (KONOS), which also operates the National Cord Blood Information Center. Until its abolition in 2024, the Statutory Cord Blood Committee deliberated on cord blood policy, eligibility criteria, and bank standards. Designated public banks also communicate with MOHW and KONOS directly and through the Korean Association of Public Cord Blood Banks, a practical consultative body without regulatory authority. The TNC threshold increase from ≥8 × 10^8^ to ≥11 × 10^8^ was implemented through formal regulatory procedures incorporating policy review, commissioned research, public notice and comment, and statutory deliberation at the time. This framework determines how empirical evidence from the present study may inform future evidence-based revisions of national standards.

Most studies have examined these factors in isolation.^12^^,^^13^^,^^17^ We aimed to: (1) quantify the impact of regulatory changes on CB banking rates; (2) evaluate the relationship between national birth trends and CB submissions; and (3) compare the effects of major infectious disease outbreaks, including potential temporal confounding with regulatory changes. Stricter criteria may improve unit quality but threaten inventory sustainability; therefore, we also assessed whether CB quality and safety were maintained throughout these disruptions.

## Methods

### Study design and setting

This retrospective study analyzed all CB units submitted to the Seoul Metropolitan Government Public Cord Blood Bank (ALLCORD) from January 2006 to December 2025; all data collection was completed prior to analysis. ALLCORD is one of four public cord blood banks authorized under the Korean Act on Cord Blood Management and Research. It undergoes biennial inspections by the Ministry of Health and Welfare and KONOS for regulatory compliance. While its procedures are benchmarked against NetCord-FACT International Standards, ALLCORD does not currently hold formal NetCord-FACT accreditation. In this study, “submissions” are defined as physical CB units received at ALLCORD from partner obstetric sites. These serve as the denominator for the banking rate (banked units/submissions); maternal registrations that did not result in a delivered unit were excluded.

The study was reviewed and granted exemption by the Institutional Review Board of Seoul National University Boramae Medical Center (IRB No. 07-2026-10), as it utilized pre-existing de-identified data with no more than minimal risk.

### Regulatory timeline and outbreak definitions

Regulatory milestones included the Cord Blood Act enactment (2010) and enforcement (2011) with TNC ≥ 8 × 10^8^ criterion—a process preceded by sustained Korean media scrutiny of private cord-blood marketing practices,^18^^,^^19^—legislative amendments following the CHA Medical Group violation (2016),^20^^,^^21^ and the revised TNC ≥ 11 × 10^8^ criterion (2021). Outbreak periods (KDCA-defined) were H1N1 (2009), MERS (2015), and COVID-19 (2020-2022). Reference periods generally spanned 2 years, except for a 3-year pre-COVID window (2017-2019) used to ensure a stable denominator for analysis. COVID-19 was subdivided into Wave 1 (2020), Waves 2-3 (2021), and Wave 4 (2022).

### CB collection, processing, and screening

Cord blood was collected by gravity into sterile collection bags (Changjo CB Collection Bag; Changjo Techno, Ansan, Republic of Korea) pre-loaded with CPDA-1 anticoagulant (registered with the Korean Ministry of Food and Drug Safety, License No. 11-1378) and processed within 36 h (transport at 4-24 °C). Processing involved hydroxyethyl starch–assisted soft-spin centrifugation and manual leukocyte-rich plasma extraction. Cryopreservation used 10% DMSO/HES cryoprotectant, controlled-rate freezing, and storage in liquid nitrogen (≤−180 °C). Banking criteria included adequate volume, prevailing TNC thresholds, negative infectious screening (HBsAg, anti-HCV, HIV Ag/Ab, syphilis, anti-HTLV, CMV IgM, sterility culture, Gram stain) performed in line with international donor screening standards,^22^ and absence of disqualifying medical history. Quality was monitored via TNC, CD34^+^ cell counts, and post-thaw viability testing.

### Statistical analysis

Segmented regression models with autoregressive error terms assessed the independent and combined effects of regulatory changes, birth rates, and outbreaks on banking metrics. Birth–submission relationships were evaluated via Pearson correlation and linear regression using national birth statistics (2006-2025) from Statistics Korea. Factor attribution quantified deviations by comparing observed banking rates against baseline trends projected from pre-regulatory periods. Categorical and continuous variables were compared using chi-square and Mann–Whitney *U* tests, respectively, and banking rate trends were evaluated with the Cochran–Armitage trend test. Two-sided *P *< .05 was significant. Analyses were performed using SPSS 26.0 and Python 3.10.

## Results

### Study population overview

**Figure 1.**
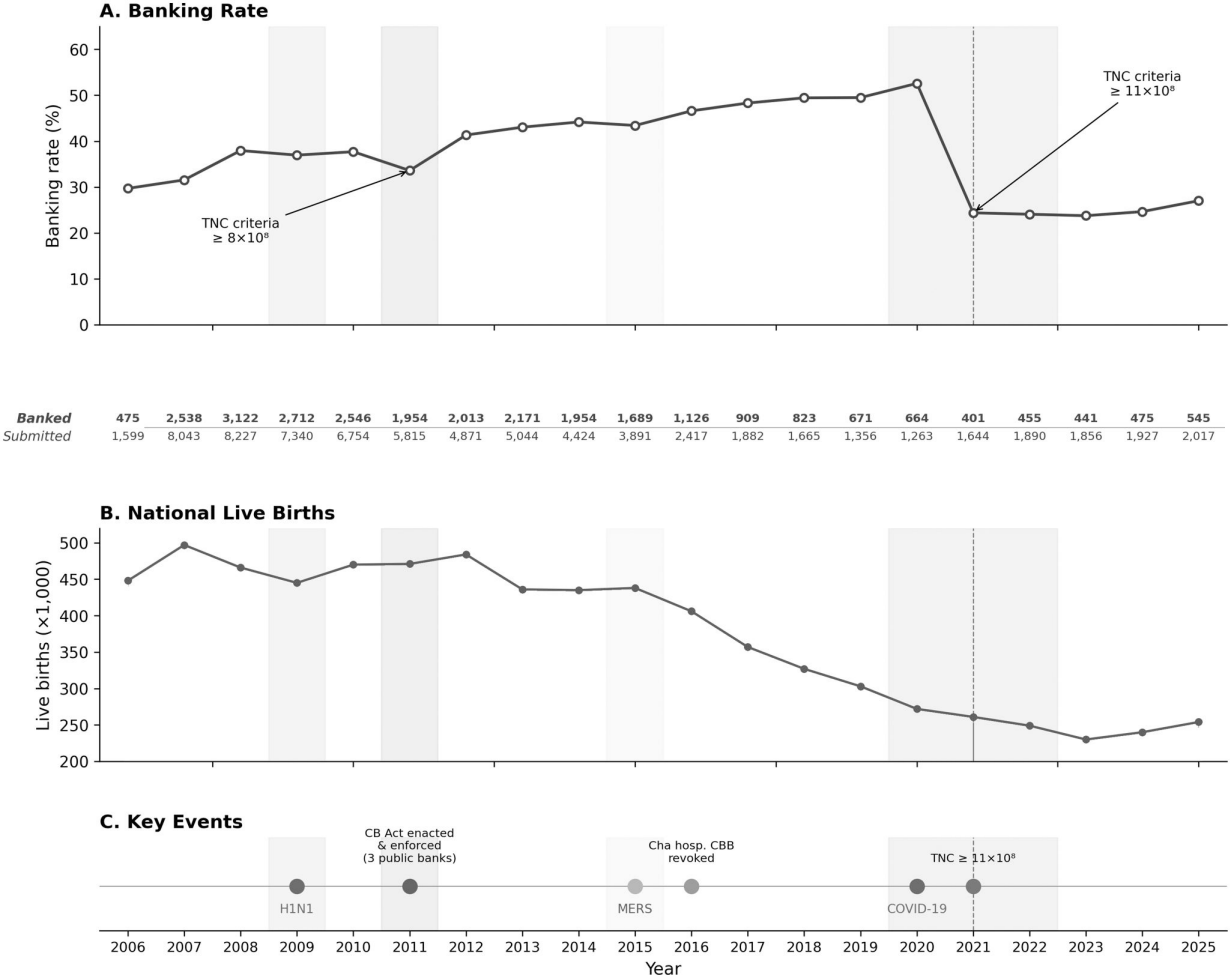
CB banking rate, national live births, and key events (2006-2025). (A) Banking rate (%) with regulatory milestone annotations including TNC criteria changes (2011, 2021); annual numbers of banked units and total submissions are listed below the panel. (B) National live births (×1000) showing progressive decline from 496 000 (2007) to 230 000 (2023), with partial recovery to 254 500 (2025). (C) Key events timeline showing regulatory milestones [CB Act enforced (2011, three public banks designated), CB bank designation revoked (2016), TNC ≥ 11 × 10^8^ (2021)] and infectious disease outbreaks (H1N1, MERS, COVID-19). Shaded regions indicate outbreak periods (H1N1, MERS, and COVID-19, each labelled in panel C); shaded regions with vertical dashed lines indicate regulatory transition points.

Of 73 925 CB units submitted over 20 years, 27 684 (37.4%) were banked, while 47.9% and 14.6% were rejected at reception and after processing, respectively. Annual submissions peaked in 2008 (8227 units), declined to 1263 units in 2020, and partially recovered to 2017 units by 2025. Banking rates fluctuated between 23.8% (2023) and 52.6% (2020; Figure 1).

### Regulatory milestones as primary drivers

**Figure 2.**
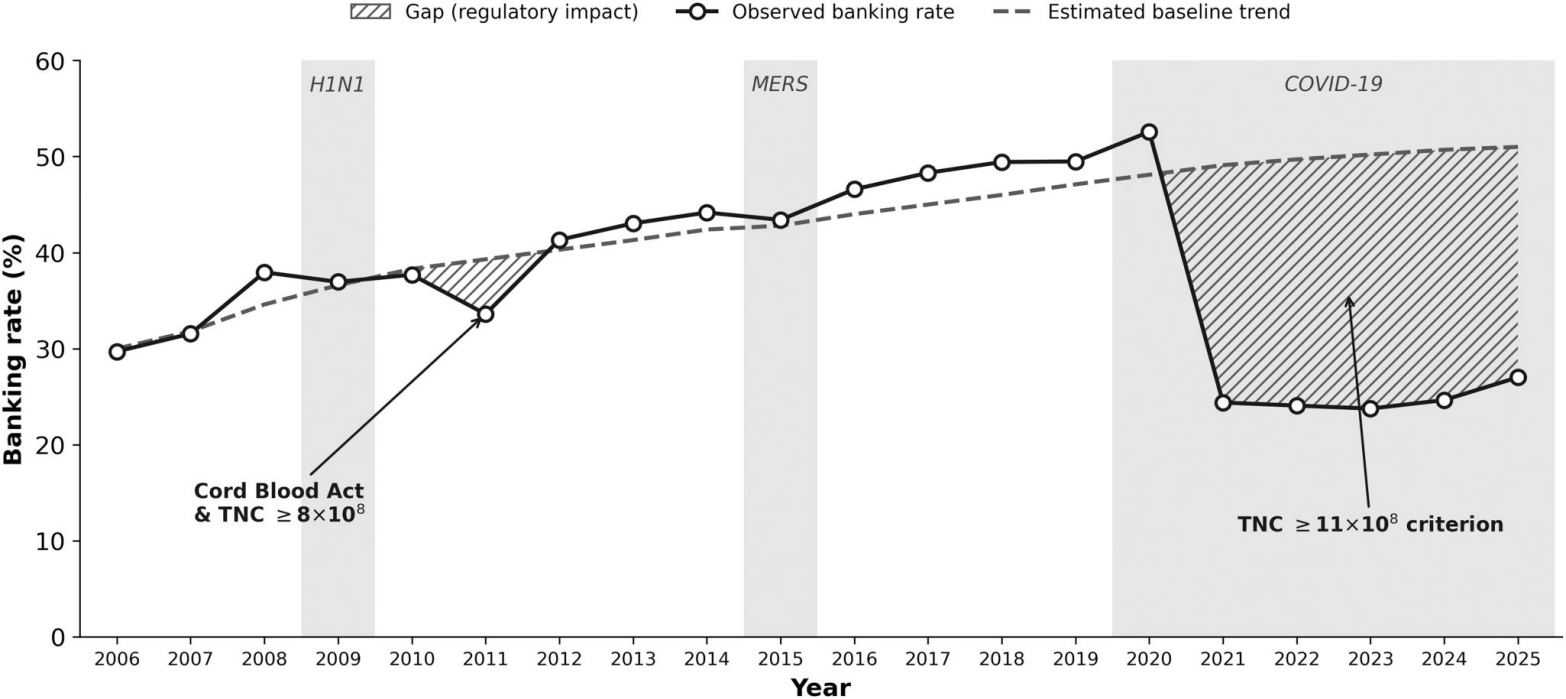
Banking rate: observed versus estimated baseline with factor attribution. The observed banking rate (solid line with open circles) is compared with an estimated baseline trend (dashed line) without regulatory interventions. Hatched areas represent the gap attributable to regulatory and outbreak effects. The two major declines (2010–2011 and 2021) are annotated with their primary drivers.

The initial TNC ≥ 8 × 10^8^ criterion (2011) produced a moderate banking rate decrease (37.7% to 33.6%), which recovered (44.2%) by 2014. However, the 2021 revision (TNC ≥ 11 × 10^8^) caused an abrupt decline from 52.6% to 24.4%, with only modest recovery (27.0%) by 2025. Interrupted time series analysis confirmed a significant level shift at the 2021 revision (*P *< .001). Factor-attribution analysis showed that the 2011 and 2021 regulatory transitions accounted for the bulk of the gap between observed banking rates and the projected baseline trend (Figure 2).

### Birth rate and number of submissions

National live births correlated strongly with CB submissions (*R*^2^ = 0.94, *P *< .001), with each 10 000-birth decrease associated with approximately 255 fewer submissions. Notably, while the shrinking donor pool reduced submission volume, it did not alter the banking rate, confirming that birth decline affected quantity rather than the quality distribution of submitted units.

### Differential outbreak impact

**Figure 3.**
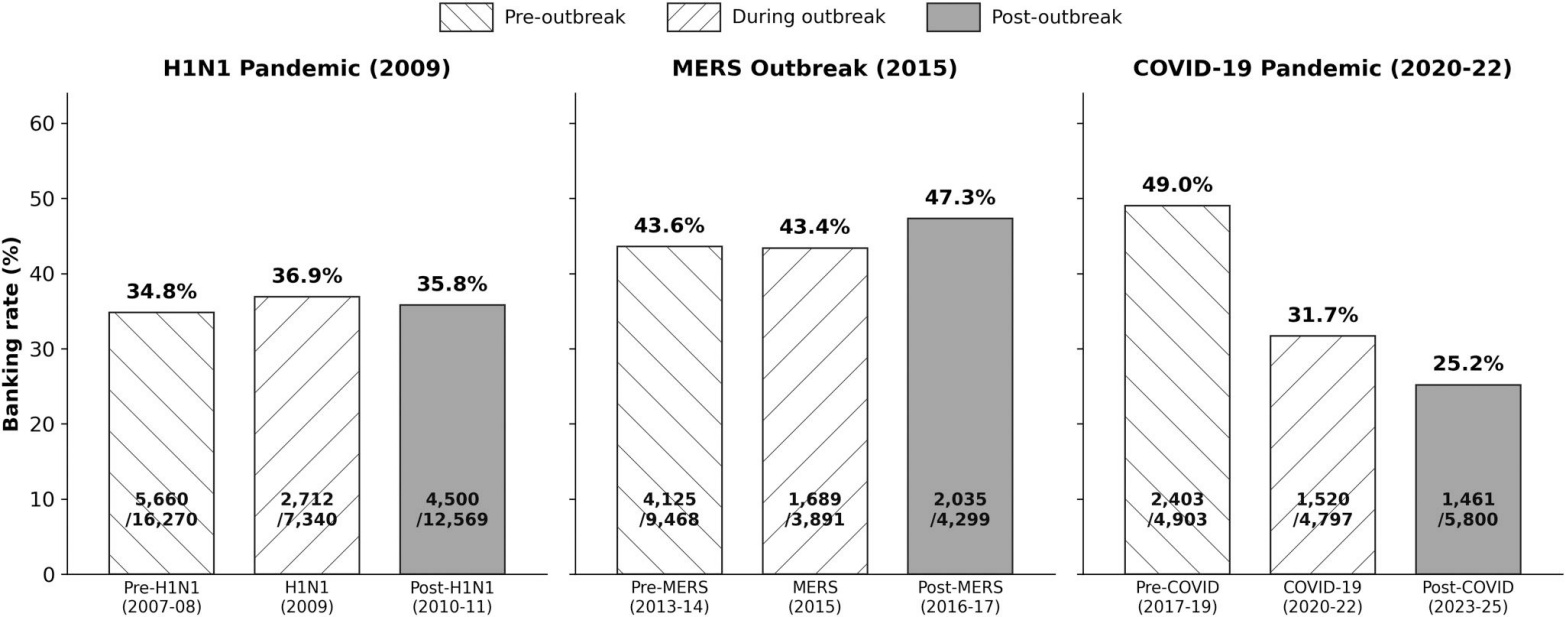
Banking rate changes across three outbreak periods. Comparison of banking rates during the pre-outbreak (left-diagonal hatching), outbreak (right-diagonal hatching), and post-outbreak (solid grey) periods for H1N1 (2009), MERS (2015), and COVID-19 (2020–2022). H1N1 and MERS showed minimal impact, while the COVID-19 period coincided with a sustained decline. The post-H1N1 decline reflects concurrent regulatory changes, not the outbreak itself. Absolute numbers of banked units and total submissions for each period are shown within the corresponding bars.

The three outbreaks showed markedly different associations with banking (Table 1; Figure 3). H1N1 and MERS had negligible effects (Δ + 2.1%p and Δ − 0.2%p, respectively), whereas the COVID-19 period coincided with a sharp 17.3%p decline relative to the pre-outbreak baseline (49.0% to 31.7%).

**Table 1:** Comparison of banking metrics across three outbreak periods.

Outbreak	Pre-outbreak period	Pre rate (%)	During outbreak	During rate (%)	Post-outbreak period	Post rate (%)	Δ Rate (%p)
**H1N1 (2009)**	2007-2008	34.8 (5660/16 270)	2009	36.9 (2712/7340)	2010-2011	35.8 (4500/12 569)	+2.1
**MERS (2015)**	2013-2014	43.6 (4125/9468)	2015	43.4 (1689/3891)	2016-2017	47.3 (2035/4299)	−0.2
**COVID-19 (2020-2022)**	2017-2019	49.0 (2403/4903)	2020-2022	31.7 (1520/4797)	2023-2025	25.2 (1461/5800)	−17.3

*Δ Rate:* change in banking rate from the pre-outbreak to the outbreak period; negative values indicate a decrease. Post-H1N1 decline reflects concurrent Cord Blood Act implementation, not H1N1 effect. The COVID-19 “during rate” (31.7%) represents the weighted average across 2020-2022; annual rates varied substantially (52.6% in 2020, 24.4% in 2021, 24.1% in 2022), reflecting the confounding effect of the 2021 TNC revision. Pre-COVID period was set to 2017-2019 (3 years) to provide a stable denominator for matched rejection-reason analysis in Table 2; H1N1 and MERS pre-outbreak windows remain 2 years by convention. Numbers in parentheses after each rate are absolute counts (banked/submissions).

### Monthly COVID-19 analysis

**Figure 4.**
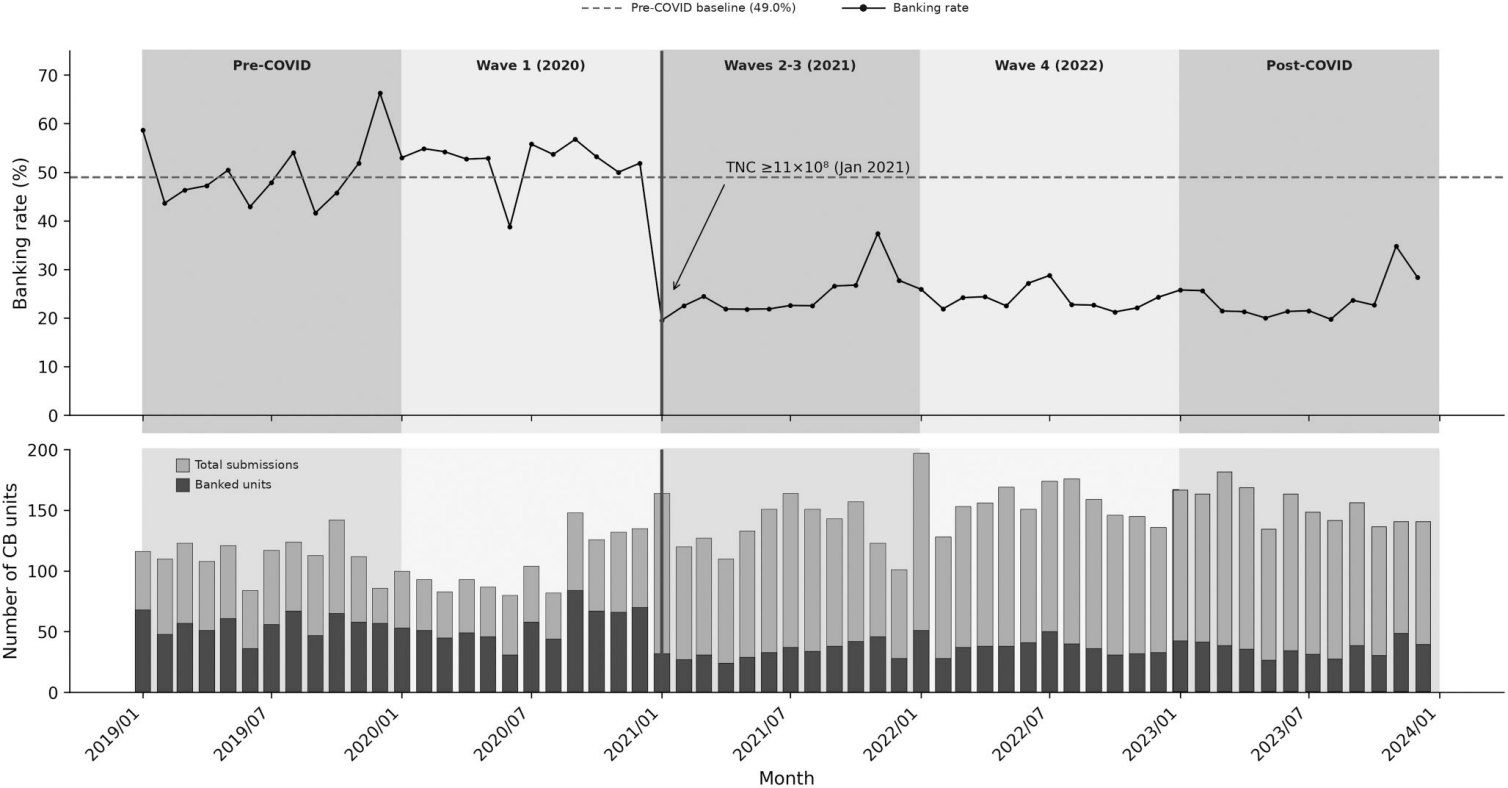
Monthly CB banking rate and number of CB units (2019-2023). Upper panel: monthly banking rate (%) with COVID-19 wave phases indicated by alternating grey shading and labelled directly on the panel. The dashed line represents the pre-COVID baseline (49.0%, pooled 2017–2019, matching Table 1). An abrupt structural break occurred in January 2021, coinciding precisely with the TNC criteria revision. Lower panel: monthly numbers of total submissions (light grey) and banked units (dark grey).

Monthly analysis (Figure 4) showed that 2020 banking rates fluctuated (30%-60%) but generally remained above the pre-COVID baseline (49.0%). However, an abrupt structural break occurred in January 2021, coinciding precisely with the TNC threshold revision. Subsequently, monthly rates consistently remained below 30%, with only modest recovery to 27.0% by 2025.

### Rejection patterns and screening

To assess the 2021 threshold impact, low-TNC rejections were stratified into 8 ≤ TNC < 11 × 10^8^ (previously bankable) versus TNC < 8 × 10^8^ (Table 2). Following the revision, units with 8 ≤ TNC < 11 × 10^8^ accounted for 28.1% (922/3277) of COVID-19 rejections and 36.3% (1574/4339) of post-COVID rejections, directly explaining more than one-third of post-2020 low-TNC rejections. Low TNC increasingly dominated rejections: 69.1% pre-COVID, 81.7% during COVID-19, and 86.4% post-COVID (*P *< .001; Table 2). Infectious disease screening positivity (HBsAg, anti-HCV, TPHA, and HIV) remained consistently low (≤0.5%) with no significant changes during outbreak periods.

**Table 2.** Distribution of rejection reasons by COVID-19 period.

Rejection reason	Pre-COVID (2017-2019) *n* = 2500	COVID-19 (2020-2022) *n* = 3277	Post-COVID (2023-2025) *n* = 4339	*P* value
**Low TNC count**	69.1	81.7	86.4	<.001
** of which TNC < 8 × 10⁸** ^a^	69.1	53.5	50.1	<.001
** of which 8 ≤ TNC < 11 × 10⁸** ^a^	0.0	28.1	36.3	<.001
**Insufficient volume**	10.1	8.6	8.4	<.05
**Time exceeded**	5.9	4.7	2.0	<.001
**Bacterial contamination**	4.4	1.4	0.8	<.001
**Processing failure**	4.0	0.5	0.3	<.001
**Maternal and family history** ^b^	3.4	0.6	0.6	<.001
**Positive ID screening**	0.6	0.5	0.5	NS
**Other/documentation**	2.5	2.0	1.0	<.001

Abbreviation: NS = not significant.

Values are percentages.

a New rows added in revision; the 8 ≤ TNC < 11 × 10^8^ category is undefined in the pre-COVID period because the prevailing threshold was ≥8 × 10^8^. After the 2021 revision, units with TNC in this band were rejected even though they would have qualified under the prior criterion (*n* = 922 in 2020-2022; *n* = 1574 in 2023-2025). Pre-COVID period (2017-2019) is consistent with Table 1.

b Maternal and family history includes review of neonatal, maternal, and family medical history (genetic, hematologic, and infectious risk factors).

### Quality of banked units

TNC count, CD34^+^ cell count, and post-thaw viability all improved progressively throughout the study period (eg, median TNC from 9.2 to 10.7 × 10^8^, CD34^+^ from 1.7 to 3.0 × 10^6^). A notable upward shift in TNC followed the 2021 threshold elevation, and quality was consistently maintained or improved during all outbreak periods.

## Discussion

In this 20-year analysis of 73 925 CB units, regulatory criteria changes—rather than infectious disease outbreaks or birth rate shifts—were the primary driver of banking rate fluctuations, demonstrated through interrupted time series analysis.

The 2021 TNC threshold revision, rather than pandemic disruption, drove the post-2020 decline: stable mean TNC values and decreased transport-time rejections during COVID-19 indicate that collection quality was maintained, although indirect pandemic effects cannot be fully excluded. While haploidentical HCT and mismatched unrelated donor transplantation have expanded donor options, BMT CTN 1101 demonstrated comparable progression-free survival between haplo-HCT and double-unit CB transplantation;^23^ earlier work in pediatric/adolescent recipients also established that single-unit CB transplantation provides outcomes comparable to double-unit transplantation.^24^ CB retains unique advantages in rapid availability, HLA diversity for under-represented populations, and as a substrate for *ex vivo–*expanded or gene-modified cellular therapies. Thus, declining public inventories should not be equated with declining clinical relevance.

Among 781 distributed units (Figure S1), median TNC was 13.6 × 10^8^; 14.3% had TNC < 10 × 10^8^ and 24.2% had TNC < 11 × 10^8^ (post-2021 threshold). These lower-dose units may remain clinically useful in Korea because of lower average adult body weight, potential use in pediatric recipients, and emerging utility as starting material for *ex vivo–*expanded or gene-modified products. Thus, the 2021 threshold likely excluded clinically meaningful units, rather than only marginal ones.

Inventory diversity, not size alone, determines HLA matching success. While all banked units undergo low-to-intermediate resolution HLA-A, -B, and -DRB1 typing, the increasing diversification of Korea’s donor population makes maintaining demographic diversity crucial under stricter thresholds. In a recent internal analysis of ALLCORD inventory, mixed or non-Korean donors accounted for approximately 12% of sampled donors and contributed additional low-frequency haplotypic diversity. ALLCORD’s current donor-engagement activities include routine recruitment through partner obstetric clinics, standardized antenatal information, periodic educational outreach events, and public donation information through the ALLCORD website. Despite this consistent engagement, the persistent submission decline suggests that routine outreach cannot offset the structural impacts of declining births and stricter regulatory policies.

The strong birth rate—submission correlation (*R*^2^ = 0.94) underscores the demographic constraint, with submissions per 1000 live births declining from 17.7 (2008) to 7.9 (2025). Whether this residual decline reflects reduced collection opportunities, waning awareness, or obstetric changes such as delayed cord clamping (DCC)—which reduces residual placental blood volume—warrants prospective investigation.

TNC threshold elevation produced a clear tradeoff: reduced banking rates in exchange for an inventory enriched with higher-quality units. Whether this improves transplant outcomes requires further study. An adaptive threshold strategy—adjusting minimum TNC criteria based on inventory levels or regional demand—could reconcile graft quality with long-term inventory sustainability.

Limitations include the single-center, retrospective design, which precludes definitive causal inference regarding the potential interaction between COVID-19 and regulatory changes. Additionally, while this study documented clinical transplantation frequency, the absence of long-term patient outcomes and engraftment success warrants further follow-up. Multicenter studies incorporating patient-level data would strengthen these findings.

In conclusion, regulatory criteria changes were the primary driver of CB banking rate fluctuations, while quality and safety were maintained throughout all disruptions. The analytical framework presented here is broadly applicable to public CB banks worldwide and supports evidence-based, data-driven threshold revisions.

## Supplementary Material

szag059_Supplementary_Data

## Data Availability

The data underlying this article cannot be shared publicly due to patient privacy regulations under the Korean Cord Blood Management and Research Act. Aggregated data supporting the findings are available within the article and its tables and figures. Requests for additional data may be directed to the corresponding author.
