# Supplementary material for: Impact of infectious disease outbreaks and regulatory policies on cord blood banking: a two-decade single-center analysis in Korea
Source: Stem Cells Transl Med. 2026 Aug 5;15(8):szag059. doi: 10.1093/stcltm/szag059 (PMC13441184; doi:10.1093/stcltm/szag059)
Supplement: Supplementary file 1 [file szag059_supplementary_data.docx]

**Supplementary Information**

# **Impact of Infectious Disease Outbreaks and Regulatory Policies on Cord Blood Banking: A Two-Decade Single-Center Analysis in Korea**

**Running head:** Outbreaks, Regulation, and CB Banking

Sue Shin^a,b,c,1^, Nam-Hee Kim^a,c,1^, Hye Ryun Lee^d^, Jong Hyun Yoon^a,b,c^, Byoung Jae Kim^b,e^, Hyun-Woong Park^a,c^, Eun Youn Roh^a,b,c,*^

^a^Department of Laboratory Medicine, SMG-SNU Boramae Medical Center, Seoul, Republic of Korea

^b^Seoul Metropolitan Government Public Cord Blood Bank (ALLCORD), Seoul, Republic of Korea

^c^Department of Laboratory Medicine, Seoul National University College of Medicine, Seoul, Republic of Korea

^d^Department of Laboratory Medicine, National Medical Center, Seoul, Republic of Korea

^e^Department of Obstetrics and Gynecology, SMG-SNU Boramae Medical Center, Seoul, Republic of Korea

^1^These authors contributed equally to this study.

***Corresponding author:** Eun Youn Roh

Department of Laboratory Medicine, Seoul National University Boramae Medical Center

20 Boramaero 5-gil, Dongjak-gu, Seoul 07061, Republic of Korea

E-mail: crimson7@snu.ac.kr

# **Supplementary Figure Legend**

**Supplementary Figure S1.** TNC and CD34^+^ profiles of 781 distributed CB units (ALLCORD, 2006–2025).

**(A)** Distribution of total nucleated cell (TNC) counts at banking for all 781 CB units distributed for transplantation between 2006 and 2025; median 13.6 × 10^8^ (interquartile range, 11.1–16.7 × 10^8^). Units are color-coded by four TNC bins: <10 × 10^8^ (red; n=112, 14.3%), 10 – <11 × 10^8^ (orange; n=77, 9.9%), 11 – <12 × 10^8^ (yellow; n=71, 9.1%), and ≥12 × 10^8^ (blue; n=521, 66.7%). Vertical dashed lines indicate the prevailing regulatory thresholds (≥8 × 10^8^, 2011; ≥11 × 10^8^, 2021). A total of 24.2% (189/781) of distributed units had TNC <11 × 10^8^ — the cell-dose range that would be deferred under the current regulatory threshold.

**(B)** Distribution of CD34^+^ cell counts at banking for the same 781 units; median 5.0 × 10^6^ (interquartile range, 3.0–7.6 × 10^6^), with the dashed line marking the median.

*Alt text: Two-panel histogram. Panel A: distribution of total nucleated cell (TNC) counts at banking for 781 cord blood units distributed for transplantation, with units color-coded by TNC bins. Panel B: distribution of CD34-positive cell counts for the same units, similarly color-coded.*

Together, panels A and B demonstrate that a clinically meaningful proportion of CB units actually used for transplantation falls below the post-2021 TNC criterion, supporting the cell-dose argument developed in the Discussion.

**Supplementary Figure S1.**
